# Prevalence and Determinants of Gestational Diabetes Mellitus: A Cross-Sectional Study in China

**DOI:** 10.3390/ijerph14121532

**Published:** 2017-12-08

**Authors:** Xianglong Xu, Ying Liu, Dengyuan Liu, Xiaoming Li, Yunshuang Rao, Manoj Sharma, Yong Zhao

**Affiliations:** 1 School of Public Health and Management, Chongqing Medical University, No. 1 Yixueyuan Road, Yuzhong District, Chongqing 400016, China; xianglong1989@126.com (X.X.); dengyuanliu@foxmail.com (D.L.); 18375750728@163.com (X.L.); rys0606@163.com (Y.R.); 2 Research Center for Medicine and Social Development, Chongqing Medical University, Chongqing 400016, China; 3 Collaborative Innovation Center of Social Risks Governance in Health, Chongqing Medical University, Chongqing 400016, China; 4 School of the Second Clinical, Chongqing Medical University, Chongqing 400016, China; lyniniy @163.com; 5 Department of Behavioral and Environmental Health, Jackson State University, Jackson, MS 39213, USA; manoj.sharma@jsums.edu

**Keywords:** gestational diabetes mellitus, prevalence, social and behavioral determinants, China

## Abstract

**Objectives:** This study aimed to identify the prevalence of gestational diabetes mellitus (GDM) and to examine its associations with social and behavioral factors, maternal body mass index (BMI), anemia, and hypertension. **Methods:** A cross-sectional analysis was performed on data collected from 2345 pregnant women from 16 hospitals in five selected provinces in mainland China. Results: Prevalence of GDM was as follows: overall: 3.7%; pregnant women in the first pregnancy: 3.4%; pregnant women in the second pregnancy: 4.6%. Compared with early pregnancy women, late-stage pregnant women were more likely to have GDM (OR = 4.32, 95% CI (1.82, 10.27)). Compared with 18–25 years old pregnant women, women aged 36–45 years were more likely to have GDM (OR = 3.98, 95% CI (1.41, 11.28). Compared with non-hypertensive patients, hypertensive patients were more likely to have GDM (OR = 6.93, 95% CI (1.28, 37.64)). However, second pregnancy, high maternal BMI, prolonged screen time (TV-viewing time, computer-using time, and mobile-phone using time), insufficient and excessive sleep duration, poor sleep quality, smoking, and secondhand smoke exposure were not significantly associated with an increased risk of GDM. **Conclusions:** Women in the second pregnancy do not appear to predict an increased risk for developing GDM than women in the first pregnancy. High-risk groups of GDM included women in their late pregnancy, aged 36–45 years old, and with hypertension. The findings will contribute to an improved understanding of social and behavioral determinants of GDM in Chinese population and contribute to the development of health-prevention promotion interventions to address GDM.

## 1. Introduction

Gestational diabetes mellitus (GDM) is associated with increased risk of adverse pregnancy outcomes, including infant macrosomia, neonatal hypoglycemia, aginal candidiasis, gestational hypertension, premature rupture of membranes, preterm birth, cesarean delivery [1,2,3,4,5], and developing type 2 diabetes 5 to 10 years after delivery [6]. Moreover, babies born from mothers with GDM have increased risk of developing impaired glucose tolerance [7]. The prevalence of GDM is rising globally [8]. Specifically in China, the prevalence of GDM increased by 2.8 times, from 2.4% to 6.8%, during 1999–2008 in urban Tianjin [9]. Previous studies in China showed that the incidence of GDM was 4.3% in 2006 [10], approximately 8.1% during 2010–2012 [11]. In addition, a previous study also found that International Association of Diabetes and Pregnancy Study Groups and American Diabetes Association criteria diagnosed 19.9% and 7.98% of women with GDM during 2012–2013 in China [12].

Social factors include maternal age, stages of pregnancy, number of pregnancy, education level, residence, and racial/ethnic disparity, behavioral factors include physical activity, sleep duration and quality of sleep, and body mass index (BMI) or pre-pregnancy overweight, family history of diabetes are associated with GDM [13,14,15,16,17,18,19,20,21,22]. In addition, women with chronic hypertension are at a higher risk of developing GDM [23]. The evidence from previous studies also suggested that prolonged screen time was associated with adverse health conditions. Adults watching more than 2 h of TV or computer daily showed a higher percentage of adverse health conditions [24,25]. Mobile phone use for more than 1 h daily was associated with a high risk of health conditions, such as parotid tumors [26]. A previous study also found that high rates of not eating breakfast and anemia were observed among pregnant women in China [27]. The factors mentioned above have been explored across many previous studies, but usually only a few factors in a study, which has provided disjointed evidence of their association with GDM. 

In 1979, the family planning policy of China was introduced to slow the population growth rate. A selective two-child policy was introduced at the end of 2013 and allowed couples nationwide to have a second child if either parent has no siblings. On 29 October 2015, the Chinese government announced a new universal two-child policy that allows all couples to have a second child. Thus, more women are likely to have a second pregnancy. However, the prevalence of GDM among pregnant women in the second pregnancy is unclear. In addition, social and behavioral determinants of GDM in China have not been studied thoroughly. Given the increasing prevalence of GDM and its adverse health outcomes, a review study indicates that the prevalence of GDM among China’s huge population suggests a substantial economic cost and further studies are needed to facilitate the prevention and treatment of GDM in China [28]. 

The aim of this study was to assess the current prevalence of GDM and to examine its associations with demographic and socio-economic characteristics, number of pregnancy, maternal BMI, behavioral factors, and disease conditions.

## 2. Methods

### 2.1. Research Methods

This study design has been reported previously [29]. In brief, all participants from 16 hospitals in Chongqing, Liaocheng, Tianjin, Chengdu, and Zunyi were invited to participate in a questionnaire survey between June and August 2015. Liaocheng and Tianjin are in north China, and Chongqing, Chengdu, and Zunyi are in south China. A total of 2345 pregnant women were included in the analysis. The study was conducted in accordance with the declaration of Helsinki, and the protocol was approved by the Ethics Committee of Chongqing Medical University (record number 2015008). All participants provided informed consent for inclusion before participating in the study.

### 2.2. Sociodemographic Variables

Socioeconomic status included age (18–25 years old, 26–35 years old, and 36–45 years old), residence (urban, rural), husband without siblings (yes, no), and marital status (married, unmarried, remarried and divorced or widowed). Number of pregnancy was categorized as first pregnancy and second pregnancy. The definition of second pregnancy is dependent on the outcome of the first pregnancy (i.e., live birth). Stages of pregnancy were classified into early pregnancy, mid-pregnancy, and late-pregnancy. Per capita monthly household income (recorded as low: <4500¥, medium: 4500–9000¥, and high: >9000¥) (1 USD = 6.575¥ in November 2017), occupation (rural migrant workers, urban and rural unemployed, unemployed, industrial workers of non-agricultural registered permanent residence, individual business, business service staff, civil servants, senior manager and middle-level manager in large and medium enterprises, private entrepreneur, professionals, clerks, students, and others), and educational level (low-primary school or junior middle school, medium-senior high school, vocational or technical secondary school and high–university) were also included.

In the multivariable analysis for GDM determinants, job condition status was categorized as manual (business services staff, rural migrant workers, and industrial workers of non-agricultural registered permanent residence), non-manual (individual business, civil servants, senior manager and middle-level manager in large and medium enterprise, private entrepreneur, professionals, clerk, and students), unemployed, and others.

## 3. Behavioral Factors

### 3.1. Routine Life

Self-reported routine life during pregnancy was categorized as regular, average, and irregular.

### 3.2. Physical Activity

Self-reported average daily physical activity time during pregnancy was asked. According to time, physical activity was categorized as insufficient and sufficient physical activity. Insufficient physical activity included daily physical activity at an average duration of less than 30 min [30].

### 3.3. Screen Time

Time spent watching TV was categorized as more than 2 h and less than 2 h [24]. Time spent on the computer was categorized as low (<2 h), medium (2–4 h), and high (>4 h) [25]. Mobile phone usage time was categorized as more than 1 h and less than 1 h [26]. 

### 3.4. Number of Meals per Day

Number of meals per day during pregnancy was categorized as <3 times, 3 times, 4 times, and ≥5 times.

### 3.5. Breakfast

Eating breakfast daily was categorized as yes and no.

### 3.6. Sleeping Duration

Self-reported hours of sleep per day during pregnancy were asked in the following manner: “How many hours did you sleep?” The question was closed and interviewees had to report the number of hours. Sleep hours were classified into insufficient sleeping duration (<7 h), sufficient sleeping duration (7–9 h), and excessive sleeping duration (>9 h) per day [31,32]. 

### 3.7. Quality of Sleep

Self-reported quality of sleep [33] was asked in the following manner: “What is your quality of sleep during the pregnancy?” Response options were very good, good, average, poor, and very poor. Therefore, during data analysis, quality of sleep was divided into three categories: very good or good, average, and poor or very poor.

### 3.8. Currently Smoking and Secondhand Smoke Exposure

Self-reported secondhand smoke exposure frequency per week [33] was divided into no exposure, daily, four to six days per week, and one to three days per week. Currently smoking [34] was defined as those who smoke during pregnancy.

## 4. BMI Category

### 4.1. BMI Group by Category of China

Participants were classified into four weight categories [35]: low weight (BMI < 18.5), normal weight (18.5 ≤ BMI < 24), overweight weight (24 ≤ BMI < 28), and obese (BMI ≥ 28). 

### 4.2. BMI Group by Category of World Health Organization

Participants were classified into low weight, normal-overweight, and obese-BMI groups as follows [36]: low weight (BMI < 18.5), normal weight (18.5 ≤ BMI < 25), overweight (25 ≤ BMI < 30), and obesity (BMI ≥ 30). 

## 5. Disease Conditions

Hypertensive patients were assessed with the following question: “Have you ever been told by a doctor or other health professional that you have hypertension (yes or no)?” Anemia and GDM were assessed using similar questions. Anemia status was categorized into anemia and non-anemia. Hypertension status was categorized into hypertension and non-hypertension.

### 5.1. Outcome Variable 

GDM status was categorized into No or Yes.

### 5.2. Data Analyses

Frequencies and percentages were calculated to summarize the distributions of categorical variables. To capture possible variables associated with the risk of GDM, univariate logistic regression analysis was utilized. Afterward, multivariate logistic regression was conducted for the following influencing factors of GDM: residence, income, trimester of pregnancy, age, mobile phone using time, maternal BMI, and hypertension. The multivariate model was statistically significant in the model coefficient test (*p* < 0.05), and obtained an acceptable fit in Hosmer and Lemeshow test (*p* > 0.5). All statistics were performed using a 2-sided test, and the statistical significance was considered at *p* < 0.05. All data analyses were performed using statistical software (SAS version 9.1.3; SAS Institute, Cary, NC, USA).

## 6. Results

### 6.1. Characteristics of Study Participants

Prevalence of GDM among all participants, women in the first pregnancy, and women in the second pregnancy were 3.7%, 3.4%, and 4.6%, respectively. Prevalence of GDM in late-pregnant women was 5.6%. Prevalence of GDM among pregnant women from rural areas was 1.7% (see Table 1).

### 6.2. Univariate Logistic Regression Analysis of Factors That Affect Gestational Diabetes Mellitus

Pregnant women in urban areas were more likely to have GDM than those in rural areas, (OR = 2.51, 95% CI (1.20, 5.22)). Compared to women with low income, women with high income were more likely to have GDM (OR = 2.05, 95% CI (1.14, 3.71)). Women in late pregnancy were more likely to have GDM than those in early pregnancy (OR = 4.25, 95% CI (1.54, 11.71)). Women aged 26–35 years old (OR = 2.33, 95% CI (1.22, 4.45)) and 36–45 years old (OR = 5.87, 95% CI (2.53, 13.62) were more likely to have GDM than pregnant women aged 18–25 years old. Compared with pregnant women of normal BMI, obese women were more likely to have GDM [BMI group by category of China: (OR = 1.97, 95% CI (1.11, 3.52), BMI group by category of World Health Organization: (OR = 2.89, 95% CI (1.42, 5.90)]. In addition, second pregnancy, prolonged screen time (TV-viewing time, computer-using time, and mobile phone-using time), insufficient physical activity, insufficient sleep duration and excessive sleep duration, poor sleep quality, smoking, and secondhand smoke exposure were not significantly associated with the prevalence of GDM (see Table 2).

### 6.3. Multivariable Logistic Regression Analysis for Gestational Diabetes Mellitus

Pregnant women aged 26–35 years old were more likely to have GDM than women aged 18–25 years old, [BMI group by category of China (OR = 1.95, 95% CI (1.01, 3.78)]. Pregnant women aged 36–45 years old were more likely to have GDM than women aged 18–25 years old, [BMI group by category of China (OR = 4.48, 95% CI (1.89, 10.67), BMI group by category of World Health Organization: (OR = 4.29, 95% CI (1.80, 10.25)]. Compared to women in early pregnancy, those in late pregnancy were more likely to have GDM [BMI group by category of China: (OR = 4.03, 95% CI (1.42, 11.41), BMI group by category of World Health Organization: (OR = 3.93, 95% CI (1.39, 11.09)]. Compared to non-hypertensive patients, patients with hypertension were more likely to have GDM [BMI group by category of China: (OR = 6.43, 95% CI (1.21, 34.16), BMI group by category of World Health Organization: (OR = 6.75, 95% CI (1.29, 35.33)]. However, no significant differences were observed between residence, income, BMI, and GDM (see Table 3).

## 7. Discussion

This cross-sectional study involved a relatively large number of pregnant women in the second pregnancy, including pregnant women in three trimesters of pregnancy: early pregnancy, mid-pregnancy, and late-stage pregnancy. In this study, the estimated prevalence of GDM among all participants, women in the first pregnancy, and women in the second pregnancy were 3.7%, 3.4%, and 4.6%, respectively. The overall prevalence of GDM was slightly lower than in a previous study conducted in 18 Chinese cities with an incidence of GDM at 4.3% [12]. We guess the possible reasons include: about seventy percent of pregnant women in this study received higher education, only one fifth of participants from rural areas and less than one in third participants of low income level. In this study, participants had a relatively high socioeconomic status. Populations in low socioeconomic status areas belong to socially vulnerable groups and those populations might be strongly influenced by chronic diseases compared with those in higher socioeconomic status areas [37]. However, second pregnancy was not significantly associated with an increased prevalence of GDM. This result is inconsistent with a previous study in the United States showed that GDM prevalence increased with the number of children [13]. However, the reasons for the difference between the two studies remain unclear. In 2015, the Chinese government introduced a new universal two-child policy that allows all couples to have a second child; thus, more women in China are likely to enter a second pregnancy in the future. A previous study also found that about half of women with GDM in their first pregnancy developed GDM in their next pregnancy [38]. Thus, prevalence of GDM among women in the first pregnancy should be controlled, and the effect of number of pregnancies on GDM in China should be identified in further study. Therefore, future studies are required to verify the relationship between number of pregnancy and GDM.

Women in their late pregnancy are more likely to have GDM than those in their early pregnancy. GDM develops when β cells fail to maintain their pace with the increasing insulin resistance that occurs during the second half of pregnancy, and the resultant increased imbalance between insulin demand and supply is manifested in the rising glucose levels [39,40,41,42]. The role of placental lactogen, which is pregnancy placenta-derived hormones, estrogen, progesterone, glucocorticoids, and placental growth hormones with insulin, includes fat decomposition that can increase the level of free fatty acids and increase pregnancy insulin resistance. In the third trimester of pregnancy, placental hormones lead to severe insulin resistance. Thus, glucose metabolism disorder occurs when the islet B cells do not compensate for the increased insulin secretion [43]. Future intervention program to reduce the prevalence of GDM should focus on pregnant women in late-pregnancy.

Pregnant women aged 36–45 years old are more likely to have GDM than women aged 18–25 years old. The result is similar to a previous study in the United States that GDM prevalence increased with maternal age [13], and a previous survey in China that the prevalence of GDM was the highest among women aged 25–34 years and the lowest among women aged 25 and under [9]. Future studies that address the age of onset and not just the age at diagnosis are important, allowing for targeted preventive measures [44]. An effective intervention program to reduce the prevalence of GDM should focus on pregnant women aged 26–35 years old and older. 

Prolonged screen time include TV viewing time, computer using time, and mobile phone using time, and physical inactivity, insufficient sleep duration or excessive sleep duration, poor sleep quality, smoking, and secondhand smoke exposure was not associated with an increased risk of GDM in this study. This result is consistent with a previous study in Massachusetts that reported no association between television viewing during pregnancy and the risk of GDM [45]. However, another study found that sitting at home for 2–4 h per day and >4 h per day was associated with increased risk of GDM [46]. And prolonged screen time was associated with developing diabetes with low energy expenditure among adult men [47] and non-pregnant women [48]. A previous study in the USA found that physical activity could reduce the risk of GDM during pregnancy and that physical activity increased insulin sensitivity via several mechanisms [45]. The findings of associations among these behaviors and GDM are inconsistent. Further studies are needed to explore the impact of these unhealthy behavioral factors during pregnancy on GDM. 

In this study, low socioeconomic status include low income, low education level, manually working and living in rural area may be not associated with increased risk of GDM. This result is in conflict with the findings of a previous study in Manitoba that not rural living is independent predictor for GDM [22]. A previous review study found that socioeconomic status was associated with health [37]. In this study, the selected hospitals are located in urban areas, and only one fifth of pregnant women from rural areas, the representative of rural pregnant women may be not very good. Further studies are required to confirm the association between these social factors and GDM. 

Maternal BMI during pregnancy may be not associated with GDM. However, a previous study in Austria among nulliparous women with a singleton pregnancy showed that obese women were at increased risk of gestational diabetes [21]. A previous review study found that overweight and obese women were at higher risk of having decreased insulin sensitivity than lean or average weight women [49]. In this study, we used the category of China and category of World Health Organization to classify BMI Group, we both found no association. We guess that maternal BMI may be not valid or appropriate to quantify obesity in pregnant Chinese women. However, future studies are required to verify this preliminary result.

The present study has some limitations, as follows: First, given that this study was a cross-sectional survey, thus, causal effects could not be established with certainty. Second, risk factors of GDM including family history of diabetes [19] and pre-pregnancy overweight status [10] were not included in this study. Third, the self-reported approach might have introduced response bias to the current findings. Self-reported previous diagnosis of disease conditions and self-reported body weight and height was obtained. Some respondents may not have answered the questions truthfully. To reduce bias, for each participant that reported disease condition, the investigator would further confirm the condition. What’s more, there may be a selection bias and the sample was not nationally representative. Only a relatively small number of rural women were included in the study. This study may be not applicable to low education level groups.

## 8. Conclusions

Estimated prevalence of GDM among all participants, women in their first pregnancy, and women in their second pregnancy were 3.7%, 3.4%, and 4.6%, respectively. Women in the second pregnancy do not appear to predict an increased risk for developing GDM. High-risk groups of GDM in pregnant Chinese women were those in late stage pregnancy, 36–45 years old, and those with hypertension. However, higher maternal BMI, prolonged screen time, insufficient sleep duration and excessive sleep duration, poor sleep quality, smoking, and secondhand smoke exposure do not appear to be associated with increased risk of GDM in this study. Further longitudinal studies are required to verify these preliminary results. The findings will improve the understanding of social and behavioral determinants of GDM among pregnant Chinese women and allow for the development of health promotion and diabetes prevention programs.

## Figures and Tables

**Table 1:** Pregnant women stratified by gestational diabetes mellitus, China (*n*, %).

Variable	Gestational Diabetes Mellitus	
	No	Yes
***Sociodemographic***		
**Number of pregnancy**		
First pregnancy	1695 (96.6)	60 (3.4)
Second pregnancy	563 (95.4)	27 (4.6)
**Nationality**		
Han nationality	2167 (96.2)	85 (3.8)
Minority	91 (97.9)	2 (2.1)
**Marital status**		
Married	2126 (96.4)	79 (3.6)
Unmarried	46 (93.9)	3 (6.1)
Remarried	66 (94.3)	4 (5.7)
Divorced or Widowed	20 (95.2)	1 (4.8)
**Education level**		
Basic education	389 (96.8)	13 (3.2)
Secondary education	342 (96.6)	12 (3.4)
Higher education	1527 (96.1)	62 (3.9)
**Residence**		
Urban area	1801 (95.8)	79 (4.2)
Rural area	457 (98.3)	8 (1.7)
**Income**		
Low	595 (97.4)	16 (2.6)
Medium	957 (96.8)	32 (3.2)
High	706 (94.8)	39 (5.2)
**Occupation**		
Rural migrant workers	116 (98.3)	2 (1.7)
Urban and rural unemployed	531 (96.0)	22 (4.0)
Industrial workers of non-agriculturalRegistered permanent residence	49 (98.0)	1 (2.0)
Individual business	189 (95.0)	10 (5.0)
Business services staff	148 (95.5)	7 (4.5)
Civil servants	388 (97.5)	10 (2.5)
Senior manager and Middle-level managerIn large and medium enterprise	92 (95.8)	4 (4.2)
Private entrepreneur	80 (91.9)	7 (8.1)
Professionals	232 (95.1)	12 (4.9)
Clerks	138 (99.3)	1 (0.7)
Students	15 (100.0)	0 (0.0)
Others	280 (96.2)	11 (3.8)
**Trimester of pregnancy**		
Early pregnant women	289 (98.6)	4 (1.4)
Mid-pregnancy women	693 (98.9)	8 (1.1)
Late-pregnant women	1276 (94.5)	75 (5.5)
**Age (years)**		
18–25	613 (98.2)	11 (1.8)
26–35	1531 (96.0)	64 (4.0)
36–45	114 (90.5)	12 (9.5)
**Behavioral factors**		
**TV viewing time per day (hours)**		
<2	1695 (96.5)	62 (3.5)
2~4	411 (96.7)	14 (3.3)
>4	152 (93.3)	11 (6.8)
**Computer using time per day (hours)**		
<2	1796 (96.4)	67 (3.6)
2~4	177 (95.7)	8 (4.3)
>4	285 (96.0)	12 (4.0)
**Mobile phone using time per day (hours)**		
<1	838 (95.4)	40 (4.6)
1~2	604 (97.1)	18 (2.9)
2~4	520 (97.0)	16 (3.0)
4~6	178 (94.7)	10 (5.3)
>6	118 (97.5)	3 (2.5)
**Physical activity**		
Insufficient	325 (97.3)	9 (2.7)
Sufficient	1933 (96.1)	78 (3.9)
**Secondhand smoke exposure**		
None exposure	1098 (96.1)	45 (3.9)
Every day	397 (97.5)	10 (2.5)
Four to six days per week	151 (96.2)	6 (3.8)
One to three per week	612 (95.9)	26 (4.1)
**Smoking during the pregnancy**		
No	2171 (96.3)	84 (3.7)
Yes	87 (96.7)	3 (33.3)
**Breakfast every day**		
No	247 (95.4)	12 (4.6)
Yes	2011 (96.4)	75 (3.6)
**Self-reported routine life during pregnancy**		
Regular	1220 (96.1)	50 (3.9)
Average	835 (96.5)	30 (3.5)
Irregular	203 (96.7)	7 (3.3)
**Number of meal per day during the pregnancy**		
3 times	1150 (96.1)	47 (3.9)
<3 times	44 (95.7)	2 (4.4)
4 times	838 (96.8)	28 (3.2)
≥5 times	226 (95.8)	10 (4.2)
**Sleep duration during the pregnancy**		
Normal sleep duration	1258 (96.8)	41 (3.2)
Insufficient sleeping duration	533 (95.0)	28 (5.0)
Excessive sleep duration	467 (96.3)	18 (3.7)
**Sleep quality status**		
Good	800 (96.7)	27 (3.3)
Average	1117 (96.3)	43 (3.7)
Poor	341 (95.3)	17 (4.8)
**BMI group by category of China**		
Normal	1050 (96.6)	37 (3.4)
Low	123 (98.4)	2 (1.6)
Overweight	826 (96.5)	30 (3.5)
Obesity	259 (93.5)	18 (6.5)
**BMI group by category of World Health Organization**		
Normal	1333 (96.6)	47 (3.4)
Low	123 (98.4)	2 (1.6)
Overweight	704 (96.2)	28 (3.8)
Obesity	98 (90.7)	10 (9.3)
**Anemia**		
No	1968 (96.1)	79 (3.9)
Yes	290 (97.3)	8 (2.7)
**Hypertension**		
No	2246 (96.4)	85 (3.7)
Yes	12 (85.7)	2 (14.3)

**Table 2:** Univariate logistic regression analysis for factors that affect gestational diabetes mellitus, China.

Variable	OR (95% CI)	*p*-Value
**Nationality**		
Minority vs. Han nationality	0.56 (0.14, 2.31)	0.423
**Marital status**		
Unmarried vs. Married	1.76 (0.53, 5.77)	0.354
Remarried vs. Married	1.63 (0.58, 4.59)	0.354
Divorced or Widowed vs. Married	1.35 (0.18, 10.15)	0.773
**Education level**		
Secondary education vs. Basic education	1.05 (0.47, 2.33)	0.905
Higher education vs. Basic education	1.22 (0.66, 2.23)	0.530
**Residence**		
Urban area vs. Rural area	2.51 (1.20, 5.22)	0.014
**Income**		
Medium vs. Low	1.24 (0.68, 2.29)	0.483
High vs. Low	2.05 (1.14, 3.71)	0.017
**Job conditions status**		
Manual vs. Non-manual	0.82 (0.41, 1.66)	0.586
Unemployed vs. Non-manual	1.07 (0.63, 1.80)	0.806
Others vs. Non-manual	1.01 (0.52, 1.99)	0.971
**Age (years)**		
26–35 vs. 18–25	2.33 (1.22, 4.45)	0.010
36–45 vs. 18–25	5.87 (2.53, 13.62)	<0.001
**Number of pregnancy**		
Second pregnancy vs. First pregnancy	1.36 (0.85, 2.16)	0.199
**Trimester of pregnancy**		
Mid-pregnancy vs. Early pregnancy	0.83 (0.25, 2.79)	0.769
Late-pregnancy vs. Early pregnancy	4.25 (1.54, 11.71)	0.005
**Self-reported routine life during pregnancy**		
Average vs. Regular	0.88 (0.55, 1.39)	0.576
Irregular vs. Regular	0.84 (0.38, 1.88)	0.674
**Smoking during the pregnancy**		
Yes vs. No	0.89 (0.28, 2.88)	0.847
**TV viewing time per day (hours)**		
2~4 vs. <2	0.93 (0.52, 1.68)	0.813
>4 vs. <2	1.98 (1.02, 3.84)	0.043
**Computer using time per day (hours)**		
2~4 vs. <2	1.21 (0.57, 2.56)	0.616
>4 vs. <2	1.13 (0.60, 2.11)	0.705
**Mobile phone using time per day (hours)**		
1~2 vs. <1	0.62 (0.35, 1.10)	0.103
2~4 vs. <1	0.65 (0.36, 1.16)	0.145
4~6 vs. <1	1.18 (0.58, 2.40)	0.654
>6 vs. <1	0.53 (0.16, 1.75)	0.299
**Physical activity**		
Sufficient vs. Insufficient	1.46 (0.72, 2.93)	0.292
**Sleep duration during the pregnancy**		
Insufficient sleeping duration vs. normal sleep duration	1.61 (0.99, 2.63)	0.057
Excessive sleep duration vs. normal sleep duration	1.18 (0.67, 2.08)	0.560
**Sleep quality status**		
Average vs. Good	1.14 (0.70, 1.86)	0.599
Poor vs. Good	1.48 (0.80, 2.75)	0.217
**Eating breakfast every day**		
Yes vs. No	0.77 (0.41, 1.43)	0.405
**Number of meal per day during the pregnancy**		
<3 times vs. 3 times	1.11 (0.26, 4.73)	0.885
4 times vs. 3 times	0.82 (0.51, 1.32)	0.407
≥5 times vs. 3 times	1.08 (0.54, 2.17)	0.823
**Secondhand smoke exposure**		
One to three per week vs. None	0.62 (0.31, 1.23)	0.170
Four to six days per week vs. None	0.97 (0.41, 2.31)	0.944
Every day vs. None	1.04 (0.63, 1.70)	0.886
**BMI group by category of China**		
Low vs. Normal	0.46 (0.11, 1.94)	0.291
Overweight vs. Normal	1.03 (0.63, 1.68)	0.904
Obesity vs. Normal	1.97 (1.11, 3.52)	0.022
**BMI group by category of World Health Organization**		
Low vs. Normal	0.46 (0.11, 1.92)	0.288
Overweight vs. Normal	1.13 (0.70, 1.82)	0.620
Obesity vs. Normal	2.89 (1.42, 5.90)	0.004
**Anemia**		
Yes vs. No	0.69 (0.33, 1.44)	0.319
**Hypertension**		
Yes vs. No	4.40 (0.97, 19.99)	0.055

**Table 3:** Multivariable logistic regression analysis for the prediction of gestational diabetes mellitus, China.

Effect	OR a	95% CI	*p*-Value	OR a	95% CI	*p*-Value
**Age (years)**						
26–35 vs. 18–25	1.95	(1.01, 3.78)	0.048	1.90	(0.98, 3.68)	0.059
36–45 vs. 18–25	4.48	(1.89, 10.67)	0.001	4.29	(1.80,10.25)	0.001
**Residence**						
Urban area vs. Rural area	1.88	(0.88, 4.04)	0.104	1.92	(0.90, 4.13)	0.093
**Income**						
Medium vs. Low	0.99	(0.53, 1.86)	0.978	0.97	(0.52, 1.81)	0.917
High vs. Low	1.35	(0.73, 2.52)	0.342	1.36	(0.73, 2.52)	0.338
**Trimester of pregnancy**						
Mid-pregnancy vs. Early pregnancy	0.81	(0.24, 2.75)	0.732	0.80	(0.24, 2.74)	0.727
Late-pregnancy vs. Early pregnant	4.03	(1.42, 11.41)	0.009	3.93	(1.39, 11.09)	0.010
**TV viewing time per day (hours)**						
2~4 vs. <2	0.87	(0.48, 1.59)	0.662	0.86	(0.47, 1.56)	0.612
>4 vs. <2	1.72	(0.87, 3.40)	0.118	1.81	(0.92, 3.57)	0.088
**BMI group**	**BMI category of China**			**BMI category of WHO**		
Lean vs. Normal	0.62	(0.14, 2.66)	0.515	0.65	(0.15, 2.79)	0.562
Overweight vs. Normal	0.70	(0.42, 1.17)	0.177	0.76	(0.46, 1.24)	0.264
Obesity vs. Normal	1.10	(0.60, 2.02)	0.755	1.67	(0.80, 3.50)	0.171
**Hypertension**						
Yes vs. No	6.43	(1.21, 34.16)	0.029	6.75	(1.29, 35.33)	0.024

a Adjusted for age, income, trimester of pregnancy, maternal BMI, and hypertension.

## References

[1] Yang X., Hsu-Hage B., Zhang H., Zhang C., Zhang Y., Zhang C. (2002). Women with impaired glucose tolerance during pregnancy have significantly poor pregnancy outcomes. Diabetes Care.

[2] Kalra P., Kachhwaha C.P., Singh H.V. (2013). Prevalence of gestational diabetes mellitus and its outcome in western Rajasthan. Indian J. Endocrinol. Metabol..

[3] Wendland E.M., Torloni M.R., Falavigna M., Trujillo J., Dode M.A., Campos M.A., Duncan B.B., Schmidt M.I. (2012). Gestational diabetes and pregnancy outcomes—A systematic review of the World Health Organization (WHO) and the International Association of Diabetes in Pregnancy Study Groups (IADPSG) diagnostic criteria. BMC Pregnancy Childbirth.

[4] Wong T., Ross G.P., Jalaludin B.B., Flack J.R. (2013). The clinical significance of overt diabetes in pregnancy. Diabet. Med..

[5] Sit D., Luther J., Dills J.L., Eng H., Wisniewski S., Wisner K.L. (2014). Abnormal screening for gestational diabetes, maternal mood disorder, and preterm birth. Bipolar Disorders.

[6] Bellamy L., Casas J.P., Hingorani A.D., Williams D. (2009). Type 2 diabetes mellitus after gestational diabetes: A systematic review and meta-analysis. Lancet.

[7] Dabelea D., Mayer-Davis E.J., Lamichhane A.P., D’Agostino R.B., Liese A.D., Vehik K.S., Narayan K.M., Zeitler P., Hamman R.F. (2008). Association of intrauterine exposure to maternal diabetes and obesity with type 2 diabetes in youth: The search case-control study. Diabetes Care.

[8] Dabelea D., Snell Bergeon J.K., Hartsfield C.L., Bischoff K.J., Hamman R.F., Mcduffie R.S. (2005). Increasing prevalence of gestational diabetes mellitus (GDM) over time and by birth cohort: Kaiser permanente of colorado GDM screening program. Diabetes Care.

[9] Zhang F., Dong L., Zhang C.P., Li B., Wen J., Gao W., Sun S., Lv F., Tian H., Tuomilehto J. (2011). Increasing prevalence of gestational diabetes mellitus in Chinese women from 1999 to 2008. Diabet. Med..

[10] Yang H., Wei Y., Gao X., Xu X., Fan L., He J., Hu Y., Liu X., Chen X., Yang Z. (2009). Risk factors for gestational diabetes mellitus in Chinese women: A prospective study of 16,286 pregnant women in China. Diabet. Med..

[11] Leng J., Shao P., Zhang C., Tian H., Zhang F., Zhang S., Dong L., Li L., Yu Z., Chan J.C.N. (2015). Prevalence of gestational diabetes mellitus and its risk factors in Chinese pregnant women: A prospective population-based study in Tianjin, China. PLoS ONE.

[12] Shang M., Lin L. (2014). I ADPSG criteria for diagnosing gestational diabetes mellitus and predicting adverse pregnancy outcomes. J. Perinatol..

[13] Desisto C.L., Kim S.Y., Sharma A.J. (2014). Peer reviewed: Prevalence estimates of gestational diabetes mellitus in the United States, pregnancy risk assessment monitoring system (prams), 2007–2010. Prev. Chronic Dis..

[14] Ranheim T., Haugen F., Staff A.C., Braekke K., Harsem N.K., Drevon C.A. (2004). Adiponectin is reduced in gestational diabetes mellitus in normal weight women. Acta Obstet. Gynecol. Scand..

[15] Solomon C.G., Willett W.C., Carey V.J., Rich-Edwards J., Hunter D.J., Colditz G.A., Stampfer M.J., Speizer F.E., Spiegelman D., Manson J.E. (1997). A prospective study of pregravid determinants of gestational diabetes mellitus. JAMA.

[16] Hunsberger M., Rosenberg K.D., Donatelle R.J. (2010). Racial/ethnic disparities in gestational diabetes mellitus: Findings from a population-based survey. Womens Health Issues.

[17] Hegaard H.K., Pedersen B.K., Nielsen B.B., Damm P. (2011). Leisure time physical activity during pregnancy and impact on gestational diabetes mellitus, pre-eclampsia, preterm delivery and birth weight: A review. Acta Obstet. Gynecol. Scand..

[18] Lao T.T., Ho L.F., Chan B.C., Leung W.C. (2006). Maternal age and prevalence of gestational diabetes mellitus. Diabetes Care.

[19] Nanda S., Savvidou M., Syngelaki A., Akolekar R., Nicolaides K.H. (2011). Prediction of gestational diabetes mellitus by maternal factors and biomarkers at 11 to 13 weeks. Prenat. Diagn..

[20] Reutrakul S., Zaidi N., Wroblewski K., Kay H.H., Ismail M., Ehrmann D.A., Van C.E. (2011). Sleep disturbances and their relationship to glucose tolerance in pregnancy. Diabetes Care.

[21] Athukorala C., Rumbold A.R., Willson K.J., Crowther C.A. (2010). The risk of adverse pregnancy outcomes in women who are overweight or obese. BMC Pregnancy Childbirth.

[22] Aljohani N., Rempel B.M., Ludwig S., Morris M., Mcquillen K., Cheang M., Murray R., Shen G.X. (2008). Gestational diabetes in Manitoba during a twenty-year period. Clin. Investig. Med..

[23] Leon M.G., Moussa H.N., Longo M., Pedroza C., Haidar Z.A., Mendez-Figueroa H., Blackwell S.C., Sibai B.M. (2016). Rate of gestational diabetes mellitus and pregnancy outcomes in patients with chronic hypertension. Am. J. Perinatol..

[24] Bowman S.A. (2006). Television-viewing characteristics of adults: Correlations to eating practices and overweight and health status. Prev. Chronic Dis..

[25] Hakala P.T., Rimpelä A.H., Saarni L.A., Salminen J.J. (2006). Frequent computer-related activities increase the risk of neck-shoulder and low back pain in adolescents. Eur. J. Public Health.

[26] Al-Qahtani K. (2016). Mobile phone use and the risk of parotid gland tumors: A retrospective case-control study. Gulf J. Oncol..

[27] Xu X., Liu S., Rao Y., Shi Z., Wang L., Sharma M., Zhao Y. (2016). Prevalence and sociodemographic and lifestyle determinants of anemia during pregnancy: A cross-sectional study of pregnant women in China. Int. J. Environ. Res. Public Health.

[28] Xu T., He Y., Dainelli L., Kai Y., Detzel P., Silvazolezzi I., Volger S., Hai F. (2017). Healthcare interventions for the prevention and control of gestational diabetes mellitus in China: A scoping review. BMC Pregnancy Childbirth.

[29] Wang L., Xu X., Baker P., Chao T., Lei Z., Qi H., Yong Z. (2016). Patterns and associated factors of caesarean delivery intention among expectant mothers in China: Implications from the implementation of China’s new national two-child policy. Int. J. Environ. Res. Public Health.

[30] The Chinese Nutrition Society (2016). The Chinese Dietary Guidelines.

[31] Petrov M.E., Lichstein K.L. (2015). Differences in sleep between black and white adults: An update and future directions. Sleep Med..

[32] Pergola B.L., Moonie S., Pharr J., Bungum T., Anderson J.L. (2017). Sleep duration associated with cardiovascular conditions among adult Nevadans. Sleep Med..

[33] Xu X., Liu D., Zhang Z., Sharma M., Zhao Y. (2017). Sleep duration and quality in pregnant women: A cross-sectional survey in China. Int. J. Environ. Res. Public Health.

[34] Xu X., Rao Y., Wang L., Liu S., Guo J.J., Sharma M., Zhao Y. (2017). Smoking in pregnancy: A cross-sectional study in China. Tob. Induc. Dis..

[35] National Health and Family Planning Commission of the People’s Republic of China Criteria of Weight for Adults. http://www.moh.gov.cn/ewebeditor/uploadfile/2013/08/20130808135715967.pdf.

[36] Elfeky O., Longo S., Lai A., Rice G.E., Salomon C. (2017). Influence of maternal BMI on the exosomal profile during gestation and their role on maternal systemic inflammation. Placenta.

[37] Feinstein J.S. (1993). The relationship between socioeconomic status and health: A review of the literature. Milbank Q..

[38] England L., Kotelchuck M., Wilson H.G., Diop H., Oppedisano P., Kim S.Y., Cui X., Shapiro-Mendoza C.K. (2015). Estimating the recurrence rate of gestational diabetes mellitus (GDM) in Massachusetts 1998–2007: Methods and findings. Matern. Child Health J..

[39] Briana D.D., Malamitsi-Puchner A. (2009). Reviews: Adipocytokines in normal and complicated pregnancies. Reprod. Sci..

[40] Ernst S., Demirci C., Valle S., Velazquez-Garcia S., Garcia-Ocaña A. (2011). Mechanisms in the adaptation of maternal β-cells during pregnancy. Diabetes Manag..

[41] Butler A.E., Cao M.L.R., Rizza R.A., Corradin A., Cobelli C., Butler P.C. (2010). Adaptive changes in pancreatic beta cell fractional area and beta cell turnover in human pregnancy. Diabetologia.

[42] Catalano P.M., Drago N.M., Amini S.B. (1998). Longitudinal changes in pancreatic beta-cell function and metabolic clearance rate of insulin in pregnant women with normal and abnormal glucose tolerance. Diabetes Care.

[43] Guo X., Wu X. (2009). Research progress of gestational diabetes mellitus. Chin. J. Diabetes.

[44] Becerra M.B., Becerra B.J. (2015). Disparities in age at diabetes diagnosis among Asian Americans: Implications for early preventive measures. Prev. Chronic Dis..

[45] Oken E., Ning Y., Rifas-Shiman S.L., Radesky J.S., Rich-Edwards J.W., Gillman M.W. (2006). Associations of physical activity and inactivity before and during pregnancy with glucose tolerance. Obstet. Gynecol..

[46] Leng J., Liu G., Zhang C., Xin S., Chen F., Li B., Tian H., Yu Z., Tuomilehto J., Hu G. (2016). Physical activity, sedentary behaviors and risk of gestational diabetes mellitus: A population-based cross-sectional study in Tianjin, China. Eur. J. Endocrinol..

[47] Rockettewagner B., Edelstein S., Venditti E.M., Reddy D., Bray G.A., Carrionpetersen M.L., Dabelea D., Delahanty L.M., Florez H., Franks P.W. (2015). The impact of lifestyle intervention on sedentary time in individuals at high risk of diabetes. Diabetologia.

[48] Hu F.B., Li T.Y., Colditz G.A., Willett W.C., Manson J.E. (2003). Television watching and other sedentary behaviors in relation to risk of obesity and type 2 diabetes mellitus in women. JAMA.

[49] Catalano P.M. (2010). Obesity, insulin resistance, and pregnancy outcome. Reproduction.

